# Molecular Packing in Network-Forming Collagens

**DOI:** 10.1100/tsw.2003.40

**Published:** 2003-06-23

**Authors:** Carlo Knupp, John M. Squire

**Affiliations:** Biological Structure and Function Section, Biomedical Sciences Division, Imperial College Faculty of Medicine, London SW7 2AZ, UK

**Keywords:** network-forming collagens, Type IV, VI, VIII, X, dogfish egg case collagen, structure, molecular assembly

## Abstract

Collagen is the most abundant protein among vertebrates and occurs in virtually all multicellular animals. Collagen molecules are classified into 21 different types and differ in their sequence, weight, structure, and function, but they can be broadly subdivided into families. Type IV, VI, VIII, X, and dogfish egg case collagens belong to the network-forming family. Here, we summarise what is known about the way these collagen molecules pack to form networks. In addition the main structural characteristics of the network-forming collagens are compared and discussed.

## COLLAGEN, A UBIQUITOUS PROTEIN

Collagen occurs in virtually all multicellular animals and is the most abundant protein among most vertebrates where it accounts for more than 25% of the total protein mass. For example, it is found in skin, cartilage, tendons, bones, teeth, cornea, placenta, lungs, heart, blood vessels, and basement membranes. It plays supportive and protective roles (in bones, tendons, placenta, etc.) and collagen aggregates are seen to be involved in cell attachment and differentiation, as chemotactic agents, as antigens and, when defective, in pathological conditions[1].

There are at least 21 different types of collagen and their amino acid sequence, molecular weight, and structure can vary considerably from one type to another.

The generally accepted definition of a collagen molecule is that it is a structural protein of the extracellular matrix that contains one or more domains having the conformation of a collagen triple helix (Fig. 1a); the collagen triple helix is characterised by the intertwining of three helical polypeptide chains (called $\alpha$ chains) of pitch 9.5 Å (Fig. 1b), forming a superhelical structure. Each polypeptide forms a left-handed helix in which every third residue comes into the centre of

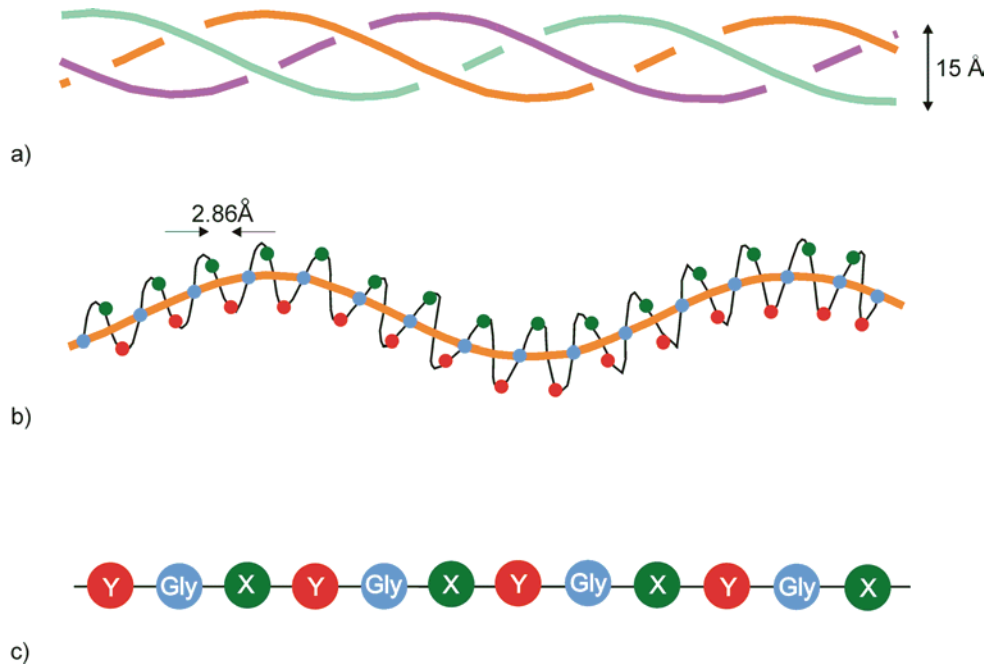

**FIGURE 1. (a)** The collagen triple helix. This helix is characterised by the right-handed intertwining of three polypeptide α-chains of pitch 9.5Å. **(b)** Each α-chain forms a left-handed helix with every third residue located in the centre of the triple helix. Here there is only space for glycines, the smallest amino acids, and there is hydrogen-bonding between the three chains. Therefore the amino acid sequence of the α-chain is characterised by the Gly-X-Y repeat **(c)**, with X and Y being often proline and hydroxyproline residues.

the superhelix. For steric reasons, the centre of the triple helix must be occupied by glycine, the smallest amino acids. Successive amino acids are separated axially by 2.86 Å. The amino acid sequence of the triple helical domains is therefore characterised by the repetition of triplets Gly-X-Y (Fig. 1c), the only sequence that does not perturb the triple helical conformation. The most frequent triplet is Gly-Pro-Hyp (10.5% of all triplets), with the imino acids Proline and Hydroxyproline contributing to the triple helix stability[2,3]. Other frequent triplets are: Gly-Leu-Hyp (5.5% of all triplets), Gly-Pro-Ala (3.4%), Gly-Ala-Hyp (3.4%), Gly-Glu-Hyp (2.8%), Gly-Pro-Lys (2.7%), Gly-Glu-Arg (2.7%), Gly-Pro-Arg (2.6%), Gly-Glu-Lys (2.5%), Gly-Phe-Hyp (2.5%), Gly-Pro-Gln (2.5%), and Gly-Ser-Hyp (2.3%). A third of the possible triplets are never observed in collagen and many triplets are rarely seen. It has been suggested that the collagen triple helical regions containing these frequent triplets are regions of high stability[3]. In the collagen triple helix, the peptide bonds linking adjacent amino acids are buried within the interior of the molecule. Thus the collagen triple helix is resistant to attack by general proteases such as pepsin. The triple helix has another important property; the amino acids in the X and Y positions have their side chains on the surface of the protein. This increases the potential for interaction and cross-links. For example, lysines and hydroxylysines are known to participate in covalent cross-linking between chains and as sites for sugar attachment[1].

Over several decades a system of nomenclature has evolved in which each type of collagen has been assigned a number, in roman numerals, generally reflecting the chronological order in which it was discovered. Further, every α-chain of a different collagen type is designated by a progressive number (α1, α2, …) followed by its collagen type number. For instance, to express the chain composition of a typical collagen I molecule, which is formed by two α1 chains and one α2 chain, the notation $[\alpha1(I)]_2[\alpha2(I)]$ is used.

Different collagen types can assemble in a wide range of structures and are classified into families depending on the structures that they form. These families can be generally classified as follows:

1. Collagens that form long fibrils with D-periodic striations every 67 nm (Type I, II, III, V, and XI collagens)
2. Fibril-associated collagens with interrupted triple helix (FACITs – Type IX, XII, XIV, XVI, XIX, XX, and XXI collagens)
3. Collagens that form networks (Type IV, VI, VIII, X, dogfish egg case collagens)
4. Collagens that form anchoring fibrils (Type VII collagen)
5. Transmembrane collagens (Type XIII and XVII);
6. Collagens with a multiple triple helical domain (multiplexins – Type XV and XVIII)

There are also other collagen types not included in this classification; for example, collagens forming the cuticle of annelids; sponging fibres; C1q Complement protein; acetyl cholinesterase; byssal thread fibres. In addition, nematocyst minicollagens have also been reported. These are the shortest collagens known with only 14 Gly-X-Y repeats and they are the main constituents of the capsule wall of the nematocyst, an explosive organelle used by cnidaria such as hydras, jellyfish, and corals for defensive, aggressive, or locomotive purposes[4,5]. Although exhaustive structural work has not yet been carried out, it is possible that these collagens are also network forming[6].

## NETWORK-FORMING COLLAGENS IN DETAIL

### Type IV Collagen

Type IV collagen is a fundamental component of basement membranes (Fig. 2a), which represent that portion of extracellular matrix that remains in direct contact with its formative cells. Basement membranes play an important role in cell adhesion, growth and differentiation, tissue repair, molecular ultrafiltration, cancer cell invasion, and metastasis.

Type IV collagen is composed of three 400-nm-long polypeptide chains. The triple helical domains of the polypeptide chains are often interrupted (Fig. 2b). In humans, for example, the α1 chain has 21 interruptions of the Gly-X-Y triplet sequence, and in the α2 chain there are 23 interruptions; overlaps between these Gly-X-Y discontinuities produce 26 irregularly spaced interruptions resulting in a collagen that is flexible[7]. The invariance of location of many of these interruptions among different species suggests a functional role[8], but few functions have yet been directly demonstrated for the interruptions. From an amino acid sequence analysis of collagen VI[9], it is apparent that interruptions of the Gly-X-Y repeat in this type of collagen reduce the stiffness of the molecule and help supercoiling. Gly-X-Y interruption may play a similar role in collagen IV. It also appears likely that the flexibility generated by the interruptions is essential to allow the formation of a network rather then a fibre[10].

Collagen IV α1 and α2 chains are found in all mammalian basement membranes[11,12]. Variant Type IV collagen α3, α4, α5, and α6 chains have been identified[13,14] and are present in a more restricted tissue distribution. The α3, α4, and α5 chains are found in the renal glomerular basement membrane and pulmonary alveolar basement membrane. The α5 and α6 chains are found in smooth muscle, aorta, bladder, mammalian duct and lobule, and epidermis basement membranes. The α3, α4, and α5 chains are thought to be responsible, when defective, for the Alport syndrome[11]. There is evidence that collagen IV molecules in basement membranes exist mainly in three forms: $[\alpha1(IV)]_2[\alpha2(IV)]$, $[\alpha3(IV)][\alpha4(IV)][\alpha5(IV)]$, and $[\alpha5(IV)]_2[\alpha6(IV)]$[11,12], although other combinations have been suggested[15,16].

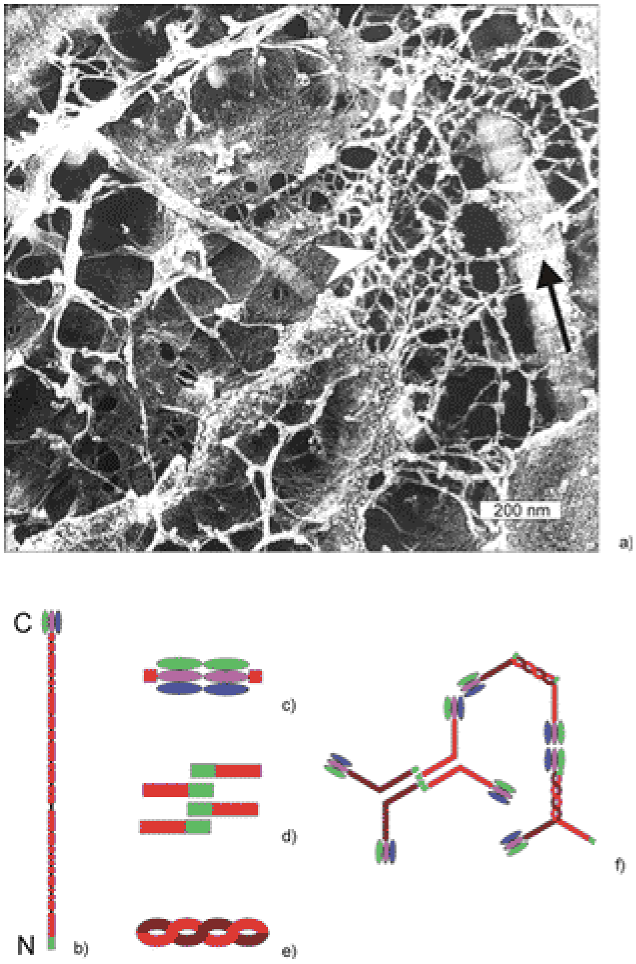

**FIGURE 2. (a)** Metal-shadowed replica of partially purified glomerular basement membrane. The arrowhead points to a collagen IV network. The black arrow indicates a Type I collagen fibre (Micrograph courtesy of Dr. M. Chew). **(b)** Diagram of a collagen IV molecule. Along the triple helical part (represented in red) numerous interruptions of the Gly-X-Y amino acid repeat are present. Linear polymerisation is observed through C-terminal interactions **(c)**, N-terminal interactions **(d)**. Lateral interactions are also observed with molecular supercoiling **(e)**. Collagen IV is able to form networks by linear and lateral associations **(f)**.

Type IV collagen can assemble into a stable three-dimensional basement membrane network via three types of interactions. In the first type, pairs of COOH-terminal globules join together to form dimers (Fig. 2c). Studies on the $[\alpha1(IV)]_2[\alpha2(IV)]$ form suggested that the dimeric globular domain is formed by disulphide exchange between corresponding cystines of the two monomeric domains[17]. It was suggested that dimerisation is an intracellular event in which the redox conditions are regulated and it may represent the first step of higher order assembly. This assembly step appears to be driven by interactions of cysteines present in the globular sequence that form the dimers by disulphide exchanges in the $\alpha1(IV)$ globular domains[18].

It has been proposed that the three different forms of collagen IV arising from the combination of the six α-chains interact at the C-terminals in a restricted way: $[\alpha1(IV)]_2[\alpha2(IV)]$ associates with another $[\alpha1(IV)]_2[\alpha2(IV)]$ or with a $[\alpha5(IV)]_2[\alpha6(IV)]$ molecule; $[\alpha3(IV)][\alpha4(IV)][\alpha5(IV)]$ associates only with another $[\alpha3(IV)][\alpha4(IV)][\alpha5(IV)]$ molecule [12,19,20].

Investigations on the $[\alpha1(IV)]_2[\alpha2(IV)]$ form indicated that in the second kind of interaction, four $NH_2$-terminal ends bind to each other to form a 28-nm end-overlapped domain (called 7S domain; Fig. 2d). Assembly proceeds through intermediates made of two or three antiparallel monomers held together through cooperative noncovalent interactions. A hydrophobic region on each $NH_2$-terminal end has been identified from the primary sequence: the maximisation of the hydrophobic contacts of the $NH_2$-terminals from four antiparallel molecules explains the antiparallel interactions with correct axial and azimuthal orientation. The complex is limited to tetramer size by the orientation arising from these interactions and cysteine and lysine/hydroxylysine residues are placed in the correct position on the corresponding chains to form disulfide and nonreducible cross-links[21]. The above two interactions can be considered as the basis for network formation.

In the third kind of interaction, Type IV collagen dimers self-interact through lateral (side-by-side) associations ([22,23,24]; Fig. 2e). This interaction has been first identified *in vitro* where assemblies showing extensive irregular networks were seen to form. The irregular polygonal geometry of the network suggests that collagen IV associates in more than one way presenting different spatial relationships. The existence of laterally associated networks (Fig. 2f) has been confirmed in tissue basement membranes of the human amnion[23]. In the electron microscope, these collagenous networks appeared as an extensive irregular polygonal network with three- to five-arm branch points, and with strands 2.5 to 7 nm in diameter. The average distance between branch points was about 45 ± 30 nm and integral globular domains, the same shape and size as those of purified collagens dimers, were also seen. Lateral associations were seen between single collagen IV filaments, with the formation of branching strands of variable diameter. Unfortunately, the localisation of the 7S domains was difficult in both the reconstructed polymers and in basement membranes, possibly because of superimposed lateral associations. The presence of supramolecular helices of collagen IV filaments was seen in amniotic networks. The similarity between reconstituted and tissue basement membrane collagen networks suggests that the information for assembly is encoded in the collagen molecules themselves. The covalent bonding of mammalian collagenous networks is formed at the amino- and carboxy-terminal regions, but the loops formed by these ends would be expected to irreversibly entrap and stabilise helically wrapped, laterally associated filaments.

## Type VI Collagen

Collagen VI is a rod-like molecule about 105 nm long with two globular domains at the extremities ([25]; Fig. 3a). It occurs mainly as a heterotrimer made of three different $\alpha$-chains, but there is evidence for the existence of less stable assemblies comprising either $\alpha$3(VI) or $\alpha$1(VI)/$\alpha$2(VI) chains. Type VI collagen microfobrils are stabilised by intra- and intermolecular disulphide bonds[26]. The $\alpha$1(VI) and $\alpha$2(VI) chains are similar in size, but the $\alpha$3(VI) chain is much larger because of a prominent $NH_2$-terminal domain. Monomers are assembled intracellularly first into antiparallel dimers and then into tetramers[26]. Purified molecules are also seen to aggregate as antiparallel dimers. The monomers associate in an antiparallel fashion with a 30-nm axial shift. Dimers consist of an inner thick rod-like region, 75 nm long, in which the two monomers are seen to intertwine four or five times, with two shorter (30 nm) and thinner rod-like segments emerging axially from the inner region ([25]; Fig. 3b). The globular ends of the two monomers are positioned at the extremities of the inner region and outer segments. Dimers can associate side by side with their 30-nm-long outer segments to form tetramers ([25,27]; Fig. 3c). Experiments carried out on cleaved N- and C-globular domains showed that they join linearly, side by side, one type connected to the other ([28]; Fig. 3d). In addition, the tetramers are seen to interact to form long chains. Dimers, tetramers, and polymeric chains have in common a 30-75-30 nm spacing between the globular ends[25,27,29].

Because of the variety of ways in which collagen VI molecules can interact with each other, they are good candidates to form networks. Lateral association of beaded filaments (likely to be collagen VI filaments) with their globular domains in register were reported as early as the 1960s[30] in association with brain tumours. More recently, more compact networks of collagen

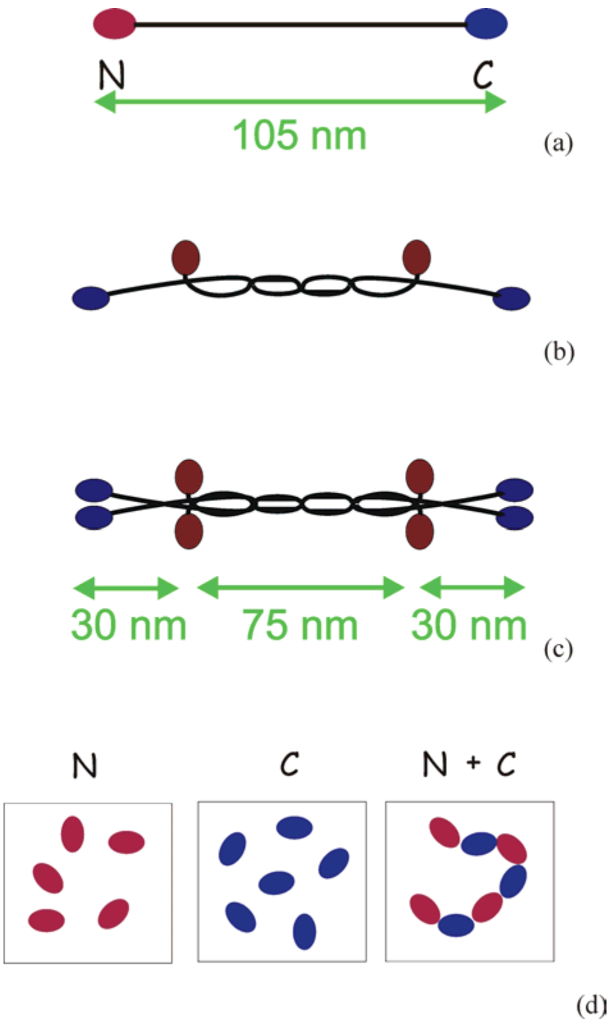

**FIGURE 3. (a)** Diagram of a collagen VI molecule. Collagen VI is a 105-nm-long molecule with massive globular domains at the extremities. Two molecules can interact laterally with a 30-nm axial shift to form dimers **(b)**. Intertwining of the monomers in the adjacent regions is seen. **(c)** Dimers join laterally to form tetramers. **(d)** Cleaved globular domains join linearly, N-terminals with C-terminals and vice versa, but homotypic N- or C-terminal associations are not seen.

VI were seen in the eyes of people suffering from Full Thickness Macular Holes ([31,32]; Fig. 4a,b), Age-Related Macular Degeneration ([33]; Fig. 4c,d), and Sorsby's Fundus Dystrophy ([34]; Fig. 4e,f). Although these ocular structures can be easily interpreted in terms of collagen VI tetramers, they differ in detail depending on the particular illness with which they are associated. Fig. 4a,b illustrates a typical appearance of the collagen structure found in the vitreous of a patient suffering from Full Thickness Macular Holes[31]. Pairs of transverse bands, 30 nm apart (arrows), are repeated axially every ~100 nm. These bands arise from the globular domains at the extremities of the collagen VI tetramer outer segments. Filaments of protein density (chevron) run axially through the pairs of bands. In some views, their lateral periodicity is about 25 nm in the bigger gap between the transverse bands, and half that much in the smaller gap between the transverse bands. The filaments are staggered laterally by half a unit cell when traversing pairs of bands. Cross-sectional views of this structure showed dots of protein density arranged on a body-centred square array of side 25 nm. The model proposed for the collagen VI assembly in these structures (Fig. 5a) consisted of collagen VI tetramers (or pairs of tetramers) interacting with four other tetramers (octamers) via their globular domains. These globular interactions involved C-

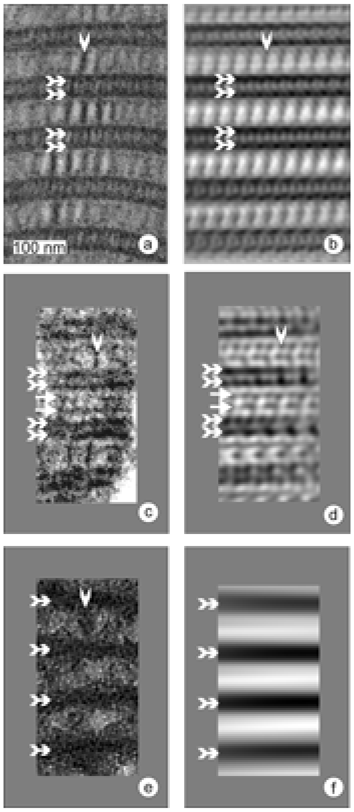

**FIGURE 4.** **(a)** Collagen VI network in the vitreous of a patient suffering from Full Thickness Macular Holes. Transverse bands 30 nm apart (double arrows, protein in black) repeat axially with a 100-nm periodicity. Axial filaments run through the bands (chevron). **(b)** Fourier-filtered image of (a). **(c)** Collagen VI network in the retina of a patient suffering from Age-Related Macular Degeneration. In addition to the structural characteristics of the aggregates described in (a), extra transverse bands of protein density run between the main pairs of bands (single arrows). **(d)** Fourier-filtered image of (c). **(e)** Collagen VI network in the retina of a patient suffering from Sorsby's Fundus Dystrophy. Here the transverse double bands are fused into a single broad band (double arrows). **(f)** Fourier-filtered image of (e).

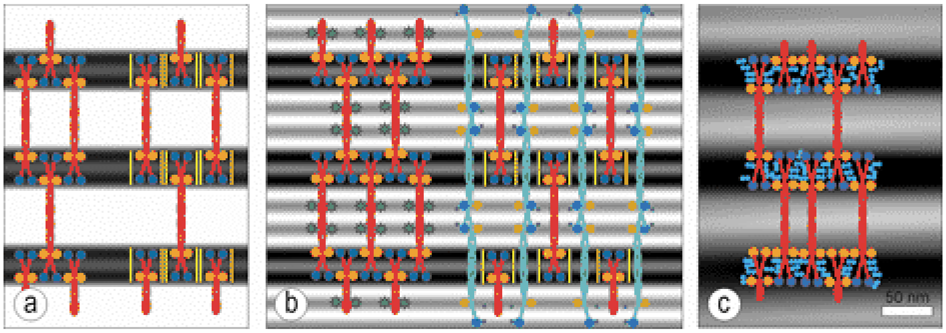

**FIGURE 5.** Models for the collagen VI tetramer packing superimposed on one-dimensional averages of the electron densities from the structures described in Fig. 4a,c, and d. Collagen VI tetramers are represented in red. N- and C-terminals are represented in blue and orange. Dimers are in light blue. **(a) Left:** Model for the assembly associated with Full Thickness Macular Holes: the packing occurs through the globular domain interactions, N-terminals interacting with the C-terminals and *vice versa.* **Right:** extra material (yellow bars) binding to the collagen VI tetramer outer segments could increase the electron density in this region. **(b)** Model for the assembly associated with Age-Related Macular Degeneration: the extra pairs of bands may arise from globular proteins binding to the tetramer triple helices (left; green stars) or more likely from the globular domains of dimers associating laterally with the tetramers (right). **(c)** Model for the assembly associated with Sorsby's Fundus Dystrophy. TIMP3 molecules (represented by blue circles) associate with the tetramer outer segments and fill the space between the bands.

terminals interacting with N-terminals and vice-versa. In doing so, the outer segments of two collagen VI tetramers belonging to two different levels lie side-by-side and give rise to the axial filaments seen in the smaller gap between pairs of transverse bands. In cross-section, the tetramer inner segments give rise to the axial filaments in the bigger gap between pairs of bands. Tetramers belonging to one level lie on a square lattice of side 25 nm. Those belonging to the next level up (or down) also lie on a square lattice having the same dimensions, but are shifted laterally by half a unit cell so that the tetramers are positioned in the centre of the square lattice in the adjacent levels. This conformation accounts for both the lateral shift of the axial filaments in the bigger gaps between bands, and the body-centred square lattice seen in transverse views. A similar arrangement can be envisaged for the collagen VI assemblies associated with Age-Related Macular Degeneration (Fig. 4c,d). In addition to the transverse bands of protein density seen in the aggregates described above, some of the assemblies associated with Age-Related Macular Degeneration present an extra pair of bands that is situated between and equidistant from the main pairs of bands (Fig. 4c,d single arrows). The profiles of main and extra pairs of bands are the same, although the extra pair is less electron dense. Models were developed to account for the secondary pairs of bands (Fig. 5b), and the preferred structure involved the presence of collagen VI dimers alongside the collagen VI tetramers. The dimers are required to be shifted axially by about 50 nm, and their globular domains would give rise to the secondary set of pairs in projection. It is likely that the dimer/tetramer association is mediated by retinal components. Extra material interacting with the tetramer outer segments, but not with those of the dimers, can account for the presence of additional mass that makes the main pairs of bands more electron dense.

In the collagen VI aggregates associated with Sorsby’s Fundus Dystrophy, extra material, analogous to that described above and tentatively identified as Tissue Inhibitor Metallo-Proteinase 3 (abundant and defective in people suffering from this disease), was found to bind to the outer segment of tetramers participating to the construction of collagen VI tetramers (Fig. 4e,f; Fig. 5c).

The different aspects that collagen VI networks present in the eyes of patients suffering from different ocular diseases suggest that collagen VI can interact with a variety of protein and retinal components (Fig. 6a–g). This characteristic is shared with collagen IV in the basement membrane, which is able to interact with a number of basement membrane components.

## Type VIII Collagen

Type VIII collagen is found in Descemet’s membrane, the basement membrane that separates corneal endothelial cells from the stroma. In addition, it is also synthesised by vascular endothelial cells and by epithelial and mesenchymal cells of other tissues[26].

Two alpha chains, α1(VIII) and α2(VIII), have been seen and it has been suggested that in Descemet's membrane the molecule exists in the $[\alpha1(VIII)]_2[\alpha2(VIII)]$ form[26,35]. However, homotrimers made of either three α1(VIII) or three α2(VIII) chains are also seen and are thermally more stable[36,37]. The variation in the chain composition of type VIII collagen may have subtle functional differences in tissues[35]. The triple helical domains of the α1(VIII) and α2(VIII) chains contain eight imperfections in the Gly-X-Y repeat. Early biosynthetic studies showed that secretion of type VIII collagen was independent of the requirement for prolyl hydroxylation, in contrast to the fibrillar collagens. In the microscope, metal-shadowed collagen VIII from cultured rabbit corneal endothelial cells showed rod-like structures (length 132 ± 5 nm) with a large knob at one end and a smaller one at the other[35].

The organisation of type VIII collagen that has been studied most closely is that in Descemet's membrane, where it forms a hexagonal lattice with electron dense nodes at the vertices

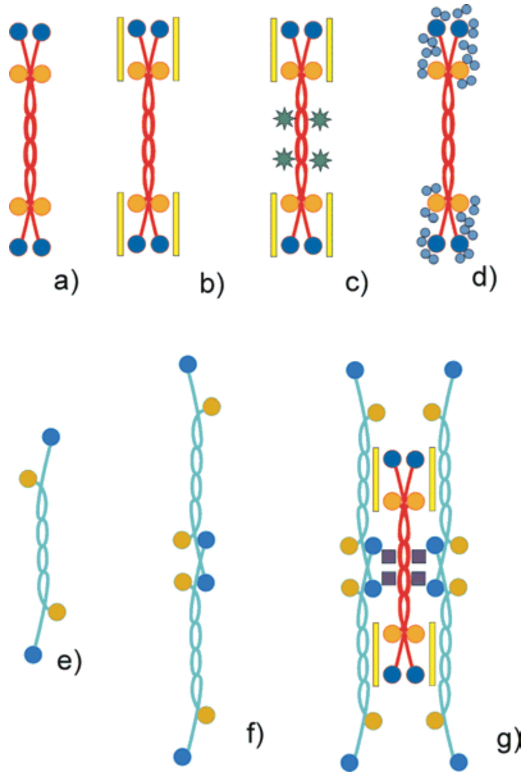

**FIGURE 6.** Summary of the possible collagen VI structural interactions that form the building blocks that make networks. **(a)** Collagen VI tetramer. **(b)** As in (a) but with extra material binding to the tetramer outer segments (yellow bars). **(c)** As in (b) but with globular proteins that may account for the formation of extra bands of protein density. **(d)** As in (a) but with TIMP3 associating with the tetramer outer segments. It may well be that TIMP3 is the extra material represented in (b). **(e)** Collagen VI dimers. **(f)** Linear interaction of two dimers through the dimer outer segments to form a chain. **(g)** Lateral associations of chains of dimers with tetramers. Extra material associating with the tetramer outer segments may explain the greater electron density seen in correspondence of the main pairs of bands.

of the hexagons, which are of side 160 ± 15 nm[35]. The hexagonal lattice structure of Descemet's membrane presumably represents a structural solution to the problem of creating a matrix that can resist compression and can maintain an open porous structure[38].

Collagen VIII appears to be involved in differentiation and tissue remodelling. It appears to be secreted by rapidly proliferating cells, it can be found in basement membranes, and it was suggested that it may act as a bridge between different types of matrix molecules[35,36,39]. Collagen VIII is present in brain, placenta, heart, lung, and thymus of embryonic and neonatal murine tissue. It is also seen in vascular tissue, arterioles and venules in muscle, heart, kidney, spleen, liver, lung, tendons, and cartilage matrix[39].

## Type X Collagen

Type X collagen is a short-chain collagen that is regulated in time and location during foetal development. It is synthesised by hypertrophic chondrocytes during enchondral bone formation and, since the extracellular matrix surrounding chondrocytes mineralises to be replaced by bone marrow and bone, it was suggested that collagen X may be associated with the mineralisation process[26,36].

Type X collagen is made of three $\alpha 1(X)$ chains. Its triple helix is about 132 nm in length, with a small $NH_2$-terminal domain and a large COOH-terminal globular domain. As in the case of collagen VIII, the triple helical domains of the α1(X) chain contain eight imperfections in the Gly-X-Y repeat. It seems to be deposited in the cartilage matrix without preprocessing[26]. *In vitro*, Type X monomers are seen to aggregate and to form multimeric clusters arranged as a lattice. The lattice consists of nodules, presumably arising from the globular domains, arranged on a hexagonal lattice and interconnected by a filamentous network formed by the triple helical parts of the collagen X molecules. The average distance between two nodules within the hexagonal lattice was about 100 ± 15 nm, a distance shorter than the measured length of 130 nm of the triple helical domain of a type X collagen molecule[40]. It was suggested that the short nodule-to-nodule distance may be in part because of the formation of superhelical structures amongst the adjacent helical domains. If this is the case, the mechanisms giving rise to supercoiling must be different from that proposed for collagen VI[9]. In collagen VI, supercoiling is driven by patches of hydrophobic amino acids coiling around the collagen triple helix. This is a direct consequence of the heterotrimeric nature of collagen VI, of which there is no evidence in collagen X, since only one α1(X) chain has been discovered so far. Lateral interactions between helical domains within individual multimeric clusters were also observed[40].

The amino acid sequence of collagen X and collagen VIII α-chains appears very similar, and hybrid molecules combining shortened α1(VIII) chains and α1(X) chains have been created[37].

## The Dogfish Egg Case Collagen

This collagen is found in the cases of the dogfish eggs. Evidence for the collagenous nature of the dogfish egg case came from X-ray diffraction studies (that indicated the existence of a 2.9 Å meridional arc), the thermal shrinkage characteristic curve (S-shaped with a mean half shrinkage temperature of 78°), and amino acid composition analysis (which showed that glycines accounted for about 16% of the amino acid residues; [41,42]). The dogfish egg case collagen was purified and observed in the electron microscope after metal shadowing. It appears to be about 45 nm long with a large globular domain at one end (about 4 nm in diameter) and a much smaller one at the other[43]. The short molecular length of the dogfish egg case collagen is thought to contribute to its ability to form liquid crystalline phases and crystalline networks. Recently, Luong and collaborators[44] sequenced, at least partially, the dogfish egg case collagen protein extracted from the nidamental gland and from newly formed eggs. From their study the dogfish egg collagen appears to contain two peptides in which repeated Gly-X-Y regions occur. The collagenous peptides, of 34 and 36 kDa, respectively, shared the same N-terminal sequence and might be two variants with different α chain composition. Homologies in sequence with Type IV and Type X collagen were recognised. Before the formation of the egg case wall the dogfish egg case collagen is stored and secreted by specialised glands[43]. The collagen assembles within the Golgi apparatus and migrates through the cytoplasm within storage granules. These undergo maturational changes as the collagen molecule aggregation state varies through different liquid crystalline phases. Finally, it flows through secretory ducts to the spinnerets where it starts to form fibrils[43,45]. The liquid crystalline changes of the collagen molecules in the storage granules are thought to be driven by pH variations generated by proton pumps and changes in the water content[43,46]. These phases have been classified by Knight et al.[45] as a poorly ordered micellar phase (Phase I), a transversely banded lamellar phase (Phase II), a cholesteric mesophase without transverse banding (Phase III), a hexagonal columnar phase (Phase IV), a second poorly oriented micellar phase (Phase V), and a highly ordered arrangement found in the mature egg case (Phase VI). *In vitro* studies carried out at pH 8, which presumably is close to the physiological pH of the egg case, generated granules showing material containing only three phases: Phase IV (hexagonal columnar; Fig. 7a), Phase VI (the mature egg arrangement; Fig. 9a),

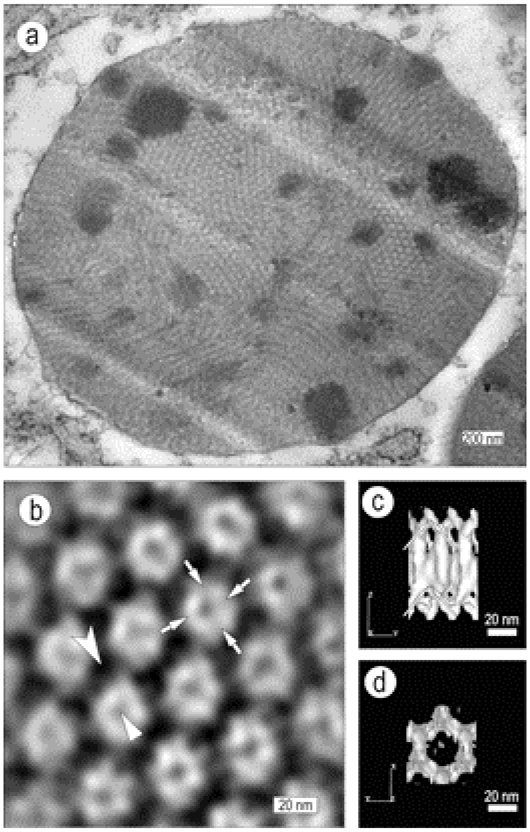

**FIGURE 7.** Hexagonal columnar liquid crystals made of dogfish egg case collagen. **(a)** Typical view of a storage granule. **(b)** Fourier-filtered image of a transverse view. The corner of each hexagon appears relatively dense and massive (chevron); a lower protein density is seen in the centre of each hexagon (arrowhead). Spikes of protein density irradiate irregularly from the centre of each hexagon (arrows). **(c)** Longitudinal view of the three-dimensional reconstruction of the hexagonal columnar collagen arrangement. Protein strands show a preferred molecular direction of about 40 to 50$^o$ to the long axis. **(d)** Transverse view. Axial columns of protein density lie on the vertices of hexagonal cells and are connected by irregular sheets of proteins.

and a new arrangement with centrosymmetrically banded fibrillar material showing a period of 17.5 nm (Phase VII)[46].

A three-dimensional reconstruction was carried out for two of these different molecular arrangements: the hexagonal columnar phase (Phase IV; [47]) and the arrangement in the mature egg case[48,49].

In the hexagonal columnar phase (Fig. 7a), the collagen presents good conformational order if seen in transverse view, but poorer order in longitudinal views. The hexagons seen in cross-section (Fig. 7b) measure about 20 nm of side. The sides of the hexagons seen in cross-section (Fig. 7b) measure about 20nm. The corners appear denser than the rest (chevron) and an area of protein density occurs in the middle of the hexagons (arrowhead). Strands of protein density are seen joining the corners and the middle of each hexagon. The reconstructed images show axial columns of protein density lying regularly on the vertices of hexagonal cells (Fig. 7c,d). These columns are connected to the three nearest neighbours by sheets of protein. Distinguishable protein strands within sheets show a preferred molecular direction of about 40 to 50$^o$ to the long axis (Fig. 7c, arrows). The packing scheme proposed for the collagen molecules in the hexagonal columnar phase was based on the interaction of the three $\alpha$-chains forming the molecule (Fig. 8a; Fig. 8c,d red molecules). The heads of three molecules join together with the tails of three other molecules presumably through hydrophobic interactions (Fig. 8a, arrows). Likely interruptions of the collagen Gly-X-Y sequence allow the molecule to kink (chevrons) and assume a 40 to 50$^{o}$ tilt to the long axis. Occasionally some molecules may

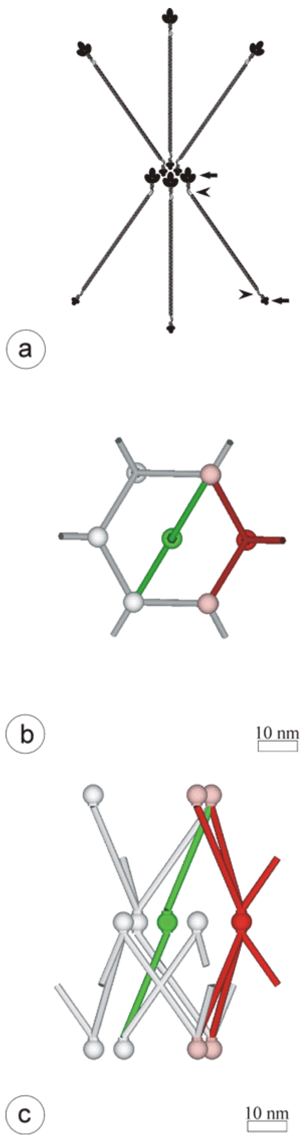

**FIGURE 8. (a)** Basic unit of the collagen arrangement in the hexagonal phase of dogfish egg case collagen. The heads of three collagen molecules join with the tails of three other molecules (arrows). Likely interruptions of the triple helices (chevrons) may endow the molecules with the necessary flexibility to form networks. **(b)** Transverse view and **(c)** longitudinal view of the molecular arrangement proposed in the hexagonal columnar phase. One basic unit is highlighted in red. Occasionally, molecules can aggregate differently (green molecules) and give rise to the protein density seen in the middle of the hexagons.

aggregate differently and cross the hexagonal unit cell giving rise to the areas of protein density in the middle of the hexagons (Fig. 8b,c, green molecules).

The arrangement of the molecules in the mature egg case (Fig. 9a) is very different from that described above. In longitudinal sections, pairs of bands of protein density about 15 nm apart are repeated axially every ~45 nm (Fig. 9b,c). Filaments of protein density run axially through the bands. In some views, the filaments that are separated laterally by 10 nm in the big gap between pairs of bands are staggered laterally by 5 nm when traversing the pairs of bands. In the small gap between bands the lateral periodicity of the filaments is 5 nm. Cross-sectional views of the egg case show that the filaments are arranged on a body-centred square lattice of side 10 nm. Consideration of the unit cell symmetry suggests that each filament in the big gap between bands is made of eight molecules, with four molecules axially shifted by ~15 nm with respect to the

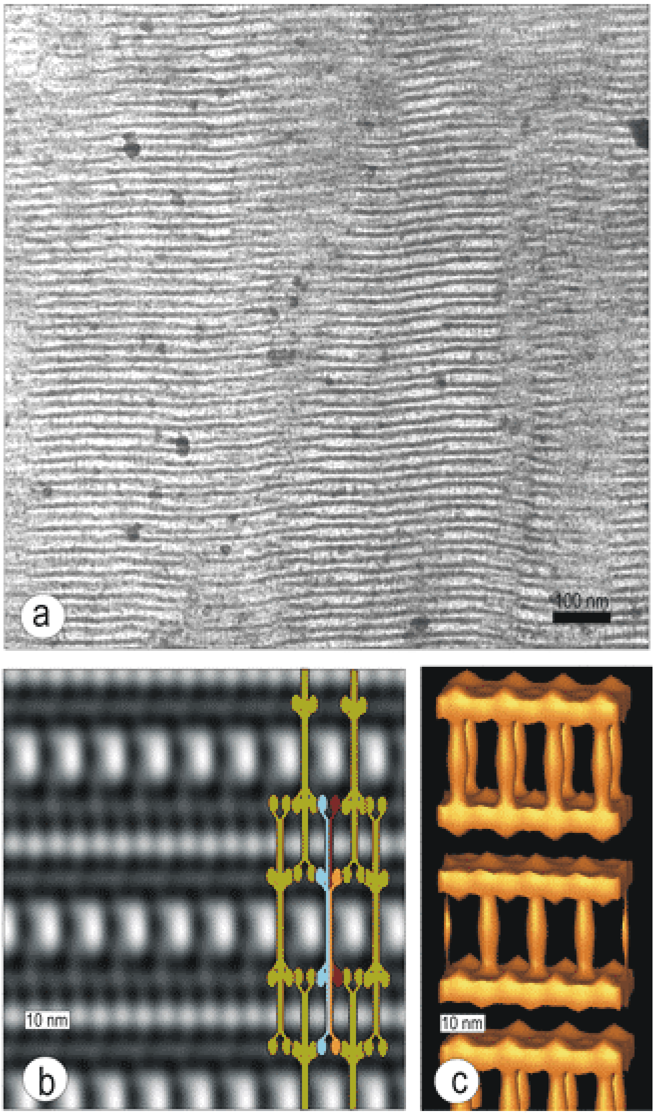

**FIGURE 9. (a)** Typical view of the collagen arrangement within the dogfish egg case. **(b)** Fourier synthesis of a longitudinal view with collagen octamers superimposed. One such octamer is colour coded to highlight single monomers (red and yellow molecules) and their axial shift. A dimer is drawn in blue. **(c)** Three dimensional reconstruction of the collagen arrangement within the egg case.

other four (Fig. 10 a,b). The globular domains at the extremities of the octamers interact with other octamer globular domains and, in projection, give rise to the transverse bands. An axial overlap of the octamers gives rise to the doubled lateral periodicity of the filaments in the small gap between the bands in longituginal view. In cross-section, groups of octamers are arranged on a square lattice of side 10 nm, and octamers belonging to different levels are laterally shifted so that the octamers at one axial level are in the middle of the square lattice of the levels above and below.

This arrangement is extraordinarily similar to that seen in the collagen VI ocular aggregates described above.

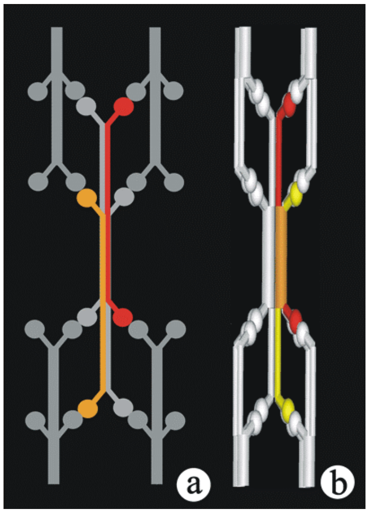

**FIGURE 10. (a)** Two-dimensional diagram of the collagen arrangement in the dogfish egg case. Two monomers are highlighted in red and yellow, and their laterally interacting parts in orange. Monomers associate laterally to form octamers, with four molecules axially shifted by 15 nm. Interactions between different octamers occur via their globular domains. (**b)** As in (a) but in three dimensions.

## Resemblance between Network Forming Collagens

The different members of the network forming collagen family vary considerably with respect to their amino acid sequence and dimensions. Nevertheless it is possible to recognise common structural patterns in the way they assemble.

Fig. 11 summarises some structural characteristics of collagen IV, VI and dogfish egg case collagen, respectively. These three kinds of collagen are all capable of associating linearly via their globular domains (Fig. 11a,b,c), and axially through their triple-helical chains (Fig. 11d,e,f).

Collagen IV associates linearly, end to end, via its C-terminals (C-terminal with C-terminal), while collagen VI and dogfish collagen appear to form this kind of association via a heterotypic aggregation of their globular domains (C-terminal with N-terminal and/or *vice versa*). Further, the three types of collagen associate laterally by antiparallel interactions. Type IV collagen is also seen to associate via its 30-nm-long N-terminals (Fig. 11c) and, in addition, by intertwining of two or more collagen molecules (Fig. 11f). Collagen VI molecules associate laterally to form antiparallel dimers and tetramers (Fig. 11e). Collagen VI monomers are axially shifted by about 30 nm when forming dimers, and they too intertwine around each other (Fig. 11e). Finally, the dogfish egg case collagen associates axially into octamers. This association involves (similarly to collagen VI) an axial displacement of four monomers by about 15 nm with respect to the other four (Fig. 11d). Although, at the resolution achieved in the structural studies of the egg case carried out so far, it was not possible to distinguish any molecular supercoiling, it cannot be excluded that some kind of molecular intertwining is formed also in this case, since the resulting structure would presumably possess a functionally desirable increase in strength.

The way collagens VIII and X assemble has not been studied in detail yet, but it appears that they too form molecular assemblies via globular interactions that involve more than two molecules. In particular, because the Type X collagen hexagonal arrangement does not account for the dimensions of the molecules (they have a smaller periodicity than expected), it was suggested that there might be axial overlapping of molecules, maybe with supercoiling. Dogfish egg case collagen forms a hexagonal structure as well and it is likely that hexagonal symmetry is a consequence of the intrinsic threefold character of the collagen molecules due to their triple helical conformation.

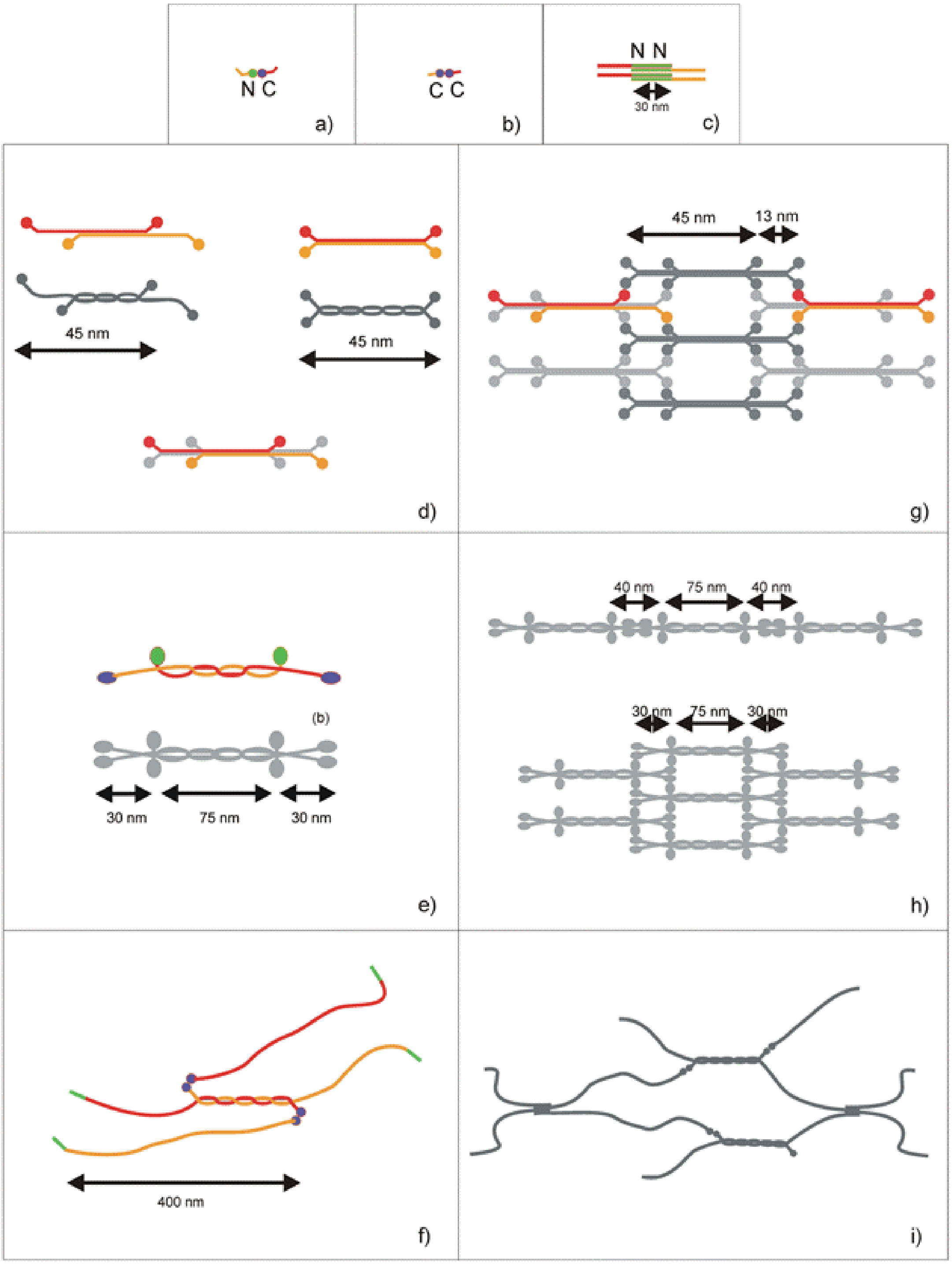

**FIGURE 11.** Summary of the structural characteristics of collagen IV, VI, and dogfish egg case collagen. These collagens can interact linearly end to end through their N- and C-terminals (represented in green and purple, respectively). Dogfish egg case and collagen VI interactions are represented in **(a)**, and collagen IV interactions in **(b)** and **(c)**. In addition, they all interact laterally to form antiparallel dimers: in **(d)** the dogfish egg collagen interactions are represented. At present, it is not known whether monomers associate with a 15-nm axial shift to form dimers, or if an axial shift occurs between already formed tetramers. Although the resolution reached does not allow a direct visualisation, it is conceivable that supercoiling occurs between monomers. Lateral interactions of collagen VI are represented in **(e)** and those of collagen IV in **(f).** Networks are formed when linear and lateral interactions take place (the dogfish egg case collagen network is represented in **(g)**, collagen VI network in **(h)** and collagen IV network in **(i)**. Collagen VI is also capable of forming long chains when tetramers associate linearly (h, top).

Linear and axial interactions of the kind described above appear fundamental in the formation of collagen networks. The interactions formed by C and N-terminals help to form linear successions of molecules, while the axial interactions are important in the generation of open networks, since they involve molecules belonging to different levels.

In addition, intertwining of different collagen molecules seems to take place in the formation of collagen networks. This behaviour is not present in other collagens (e.g., collagen I). Interruptions in the Gly-X-Y sequence of the network forming collagen are a common characteristic. This may help reduce the stiffness of the collagen molecule, allowing more spatial freedom for the molecule, and also promoting supercoiling.

Finally, collagen IV and VI seem to be able to interact with other molecules and proteins. It is probable that this characteristic is used in strengthening the network formed by these collagens, as well as having other physiological implications.

## ACKNOWLEDGEMENTS

The authors wish to thank the Wellcome Trust for their financial support (Grant # 389048)
